# Inherited KIF21A and PAX6 gene mutations in a boy with congenital Fibrosis of extraocular muscles and aniridia

**DOI:** 10.1186/1471-2350-14-63

**Published:** 2013-06-21

**Authors:** Ming Ying, Ruifang Han, Peng Hao, Liming Wang, Ningdong Li

**Affiliations:** 1 Tianjin Eye Hospital, Tianjin, 300022, PR China; 2 Tianjin Key Lab of Ophthalmology and Visual Science, Tianjin, PR China; 3 Tianjin Eye Institute, Tianjin, PR China; 4 Clinical College of Ophthalmology, Tianjin Medical University, Tianjin, PR China

## Abstract

**Background:**

Mutations in the *KIF21A* gene are detected in the patients with congenital fibrosis of the extraocular muscles. Mutations in the *PAX6* gene are detected in the patients with congenital aniridia.

**Case presentation:**

Herein we report a boy with both congenital fibrosis of extraocular muscles and aniridia. Sequence analysis of his *KIF21A* and *PAX6* genes reveals a 1-bp deletion (c.745delC) in the PAX6 gene and a missense mutation of c.2860C > T (p.Arg954Trp) in *KIF21A*.

**Conclusions:**

This study demonstrates that the occurrence of independent mutations in more than a single gene in a patient may lead to a complex phenotype.

## Background

Congenital fibrosis of the extraocular muscles (CFEOM) is a clinically and genetically heterogeneous group of ocular motor diseases characterized by congenital restrictive ophthalmoplegia affecting extraocular muscles innervated by the oculomotor and/or trochlear nerves [1]. Three types of CFEOM have been defined according to the inheritance pattern and clinical features. CFEOM1 (OMIM,135700) is an autosomal dominant disorder with complete penetrance and caused by the mutations in the KIF21A gene (OMIM,608283) on chromosome 12q12. Individuals with CFEOM1 have typical clinical phenotypes including bilateral blepharoptosis, chin elevation and downward fixation of both eyes without ability to elevate above the horizontal midline. CFEOM2 (OMIM,602078) is an autosomal recessive disorder caused by mutations in the ARIX gene (OMIM, 602753) on chromosome 11q13. The affected individual shows bilateral ptosis and ophthalmoplegia with the globes “frozen” in an extreme abducted position. CFEOM3 is an autosomal dominant disorder with incomplete penetrance and shows variable clinical features. Affected individuals may have unilateral or bilateral restrictive ophthalmoplegia with or without blepharoptosis and can raise their eyes above midline. CFEOM3 has been further sub-categorized into CFEOM3A, CFEOM3B and CFEOM3C. CFEOM3A (OMIM, 600638) is caused by mutations in the TUBB3 gene (OMIM, 602661) on chromosome 16q24, and CFEOM3B (OMIM, 608283) is caused by mutations in the KIF21A gene. CFEOM3C (OMIM,609384) has been mapped to chromosome 13q.

Congenital aniridia (OMIM,106210) is a rare ocular malformation that affects the development of multiple ocular structures. The most noticeable sign of this disorder is complete or partial iris hypoplasia with associated foveal hypoplasia, resulting in reduced visual acuity and nystagmus presenting in early infancy. Other ocular anomalies, such as corneal opacity, cataract, glaucoma, coloboma and optic nerve hypoplasia, are also frequently associated with aniridia [2]. Aniridia may occur either as an isolated ocular abnormality without systemic involvement, caused by mutations in the Paired Box gene 6 (PAX6) located on chromosome 11p13, or as a part of the Wilms tumor-aniridia-genital anomalies-retardation (WAGR) syndrome (OMIM,194072), caused by deletion of a region of chromosome 11p13-p12 containing both the Wilms tumor 1 (WT1) and PAX6 genes. In addition, PAX6 gene mutations can be associated with brain malformations [3].

Here we report a male proband with aniridia and CFEOM1. He was determined to have inherited a KIF21A gene mutation from his mother who is the daughter and sister of individuals with CFEOM1. In addition, the proband inherited a mutation in PAX6 from his father, who also had congenital aniridia.

## Case presentation

A 7-year old male (individual III:1, Figure 1A) with both CFEOM1 and congenital aniridia was recruited as were his family members, including his parents, grandfather (individual I:1), grandmother (individual I:2), uncle (individual II:3) and aunt (individual II:4). He was found to have no ability to make pursuit movements soon after birth. Ophthalmologic examinations at age 2 revealed that he had congenital fibrosis of the extraocular muscles, aniridia, foveal hypoplasia, and nystagmus, but had normal corneas and transparent lenses.

**Figure 1:**
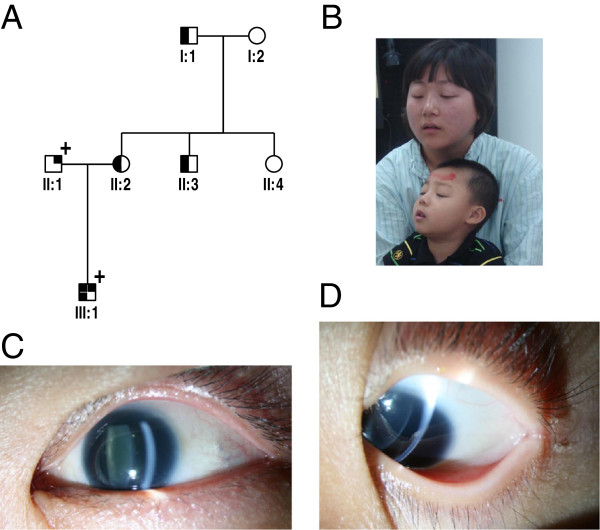
**Phenotypes of the affected boy and his parents.** (**A**) The pedigree, showing the boy inheriting CFEOM from his mother and aniridia from his father. (**B**) The boy and his mother, both of them with typical clinical features of CFEOM1. (**C**) The photo of the anterior segment in the left eye of the boy’s father, showing aniridia. (**D**) Aniridia in the left eye of the boy.

His grandfather (individual I:1) was the first individual in this pedigree to have congenital fibrosis of extraocular muscles. The grandfather was found to have limited extraocular movement soon after birth and underwent strabismus surgery for treatment of CFEOM in 1991. His head posture and ocular movement had been improved after surgery. The boy’s mother underwent strabismus surgery for CFEOM treatment in 2003. Her head posture and eye position were improved after surgery. His uncle (individual II:3) was diagnosed with CFEOM, but has not undergone strabismus surgery. All affected individuals with CFEOM in this family have typical features of CFEOM1 including bilateral blepharoptosis, chin elevation and ophthalmoplegia with the eyes fixed in an infraducted position of roughly 20 to 30 degrees below the horizontal midline (Figure 1B).

The proband’s father was affected with congenital aniridia, foveal hypoplasia and nystagmus. The iris was completely absent at its base in both of his eyes, similar to the iris in the boy’s eyes (Figure 1C, 1D). He had normal corneas and transparent lenses with the best corrected visual acuity of 0.1 in both of his eyes. In addition to an ocular examination, systemic and neurological examinations were also performed for all affected individuals in this family. For differentiating isolated aniridia from WAGR syndrome, abdominal ultrasonographic assessments were performed in each of the affected individuals with aniridia. However, no abnormal signs in the kidney or urogenital system were detected by B-scan, and neither were mental retardation, cerebellar anomaly, and olfactory or hearing difficulties found in the boy and his father as well.

## Method

### Mutation analysis

This study was approved by the Ethics Committee of Tianjin Eye Hospital and was conducted in accordance with the Declaration of Helsinki. After the written informed consent was obtained from all participants, 3 ml blood samples were taken from each individual and DNA was extracted from blood lymphocytes, according to the standard methods of protocol (Roche Biochemical, Inc).

The sequence analysis of PAX6 and KIF21A genes was performed by direct DNA sequencing. All coding regions of the PAX6 and KIF21A genes were amplified by polymerase chain reaction (PCR) from genomic DNA. Primers for all coding exons and exon-intron boundaries of the target genes were designed by the Primer3 program (http://frodo.wi.mit.edu/). All PCR primer sequences are not shown but can be available on request. Amplifications were carried out in 50 μL of standard PCR buffer containing 1.5 mM MgCl2, 0.2 mM of each dNTP, 0.5 μM of each primer, 1 U of Taq polymerase, and 50 ng of DNA. The amplification program was an initial 2 min denaturation at 98°C, followed by 30 cycles of 30 s at 94°C, 30 s at 55°C, 1 min at 72°C, and a final 7 min extension step at 72°C. The PCR products were extracted using the QIAquick Gel Extraction Kit (Qiagen, Valencia, CA). DNA sequencing analysis was performed using the BigDye Terminator Cycle Sequencing V3.1 kit on an ABI PRISM 3130 Genetic Analyzer (Applied Biosystems). PCR products of heterozygous mutants were ligated to pMD®18-T vectors (Sagon, Shanghai, China), and subsequently sequenced by the 3130 Genetic Analyzer. Sequencing results were assembled and analyzed with the Seqman program of DNASTAR software (DNASTAR Inc, Madison, WI). The referred standard sequence of the PAX6 and KIF21A genes are from the GenBank (NM_001173464 for KIF21A, NM_000280 for PAX6). The cDNA numbering +1 corresponds to A in the ATG translation initiation codon in the reference sequence. Mutation naming followed the nomenclature recommended by the Human Genomic Variation Society (HGVS).

## Results

**Figure 2:**
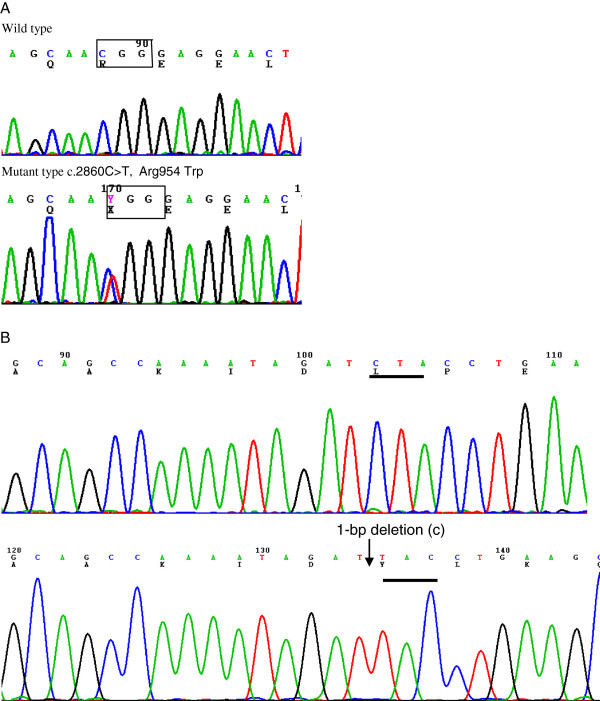
**Heterozygous mutations in the KIF21A and PAX6.** (**A**) A C to T change at 2860 nt in exon 21 of the KIF21A resulting in Arginine (**A**) at condon 954 substituted by Tryptophan (W); (**B**) A 1-bp deletion in exon11 of PAX6 gene was detected by cloning sequencing, which resulted in a frameshift mutation (p.Leu249TyrfsX22).

Since the proband inherited the phenotype of CFEOM1 from his mother, his KIF21A gene was sequenced. After all coding regions of KIF21A gene were sequenced, a heterozygous c.2860C > T (p.Arg954Trp) mutation was found in exon 21 of KIF21A gene (Figure 2). In addition, the c.2860C > T (p.Arg954Trp) mutation was also detected in all affected family members with CFEOM (individual I:1, II:2, and II:3) but was absent in individual I:2, II:1, and II:4, as well as in 100 unaffected Chinese control individuals.

Sequencing of PAX6 showed a heterozygous 1-base pair deletion (c.745delC, p.Leu249TyrfsX22) in exon11 in the proband and his father. This results in a frameshift change in PAX6 predicted to produce a truncated protein with the initial 248 amino acids similar to the wild type PAX6 protein and 22 additional aberrant amino acids followed by a premature stop codon. Thus, the truncated mutant PAX6 protein is missing the final 152 amino acids of wild type PAX6 protein (Figure 2).This change was absent by sequencing in 100 Chinese male normal controls.

## Discussion

In this study, we report the clinical features of a 7 year old male with both CFEOM1 and congenital aniridia. He inherited the c.2860C > T (p.Arg954Trp) mutation in KIF21A from his mother, who also had CFEOM1, and the c.745delC (p.Leu249TyrfsX22) mutation in PAX6 from his father who had congenital aniridia.

The KIF21A gene comprises 38 exons and encodes a 1674 amino acid protein. Structurally, the KIF21A protein includes an N-terminal motor domain, a coiled-coil domain, and a C-terminal WD-40 repeat domain [4]. Functionally, the KIF21A protein is responsible for the transport of membranous organelles, protein complexes, and mRNAs to specific destinations within the cell in a microtubule-and ATP-dependent manner. These are essential for normal morphogenesis and functioning of the cell [5]. To date, 13 mutations in the KIF21A gene are reported to be detected in the affected individuals with CFEOM, of which 12 are missense mutations [4,6-9].

The c.2860C > T (p.Arg954Trp) missense mutation in exon21 is the most common in the KIF21A gene and accounts for 84% of KIF21A mutations seen in CFEOM [4]. It has been detected in different ethnic populations, including European, African, Arabian, and Asian [4]. R954 is located in the coiled-coil domain which comprises a repeated heptad consensus sequences, and is regarded as an important site for KIF21A function including modulating the assembly and stability properties of the protein structure and interaction. Three mutations at Arg954 (p.Arg954Trp, Arg954Gln and Arg954Leu) [4] were identified in the patients with CFEOM. The recurrent mutation of p.Arg954Trp in our study further supports the idea that R954 is a mutation “hotspot” in the KIF21A.

PAX6 is a member of the paired box gene family and encodes a transcriptional regulator that recognizes target genes through its highly conserved paired-box and homeobox domains [10]. The human PAX6 gene consists of 14 exons and may produce at least three transcript isoforms by alternative splicing. The p.Leu249TyrfsX22 mutation in this study is located in the homeodomain and caused by a 1-bp deletion which results in a frameshift mutation and would be predicted to produce a truncated protein with the initial 248 amino acids similar to the wild type PAX6 protein followed by an additional 22 aberrant amino acids and then a premature termination codon (PTC). In mammalian cells, mRNAs containing a PTC due to a nonsense mutation or a frameshift mutation in an internal exon are usually recognized and degraded by nonsense-mediated mRNA decay (NMD), which is a preventive mechanism for eliminating the production of potentially deleterious truncated proteins interfering with normal cellular processes [11]. As a general rule, mammalian transcripts that contain a PTC more than 50–55 nucleotides (nt) upstream of the last exon-exon junction will be subjected to NMD, while PTCs located within 50–55 nt or downstream of the last exon-exon junction are not recognized by NMD and a truncated protein may be formed. The p.Leu249TyrfsX22 mutation occurred in exon11 of the PAX6 gene and produced a PTC 369 nucleotides distant from last exon-exon junction, and would be predicted to be recognized by the NMD mechanism.

Functionally, the PAX6 protein is critical for the development of various tissues and organs, particularly the eye and the nervous system. Mutations in the PAX6 gene may cause aniridia, coloboma, hereditary keratitis (KERH, OMIM:148190), bilateral optic nerve hypoplasia [12] (BONH), interhemispheric brain malformations [3], Peters syndrome (PAN, OMIM 604229), Gillespie syndrome (OMIM:206700), and probably myopia [13]. Mutations in the PAX6 have not yet been reported to be associated with congenital fibrosis of extraocular muscle, but may be associated with congenital ptosis [14].

The boy in this study has the clinical features of CFEOM1 and congenital aniridia. By DNA sequence analysis, we found that he inherited the p.Arg954Trp mutation in the KIF21A from his mother and the p.Leu249TyrfsX22 mutation from his father. Although PAX6 mutations may cause the phenotype of congenital ptosis, comparing the feature of ptosis with his mother, we didn’t find his bilateral ptosis was more serious than his mother’s, which suggested that his bilateral ptosis with ophthalmoplegia was caused by the KIF21A mutation. As an offspring in the family with both CFEOM1 and aniridia, the boy has 50% risk to be an affected individual with either CFEOM1 or aniridia, and would be 25% risk of developing both CFEOM1 and aniridia. The probability of developing both CFEOM1 and aniridia for their additional child would be 25%, should his parents produce an additional child. However, since his aniridia is a consequence of an internal mutation within the PAX6 gene rather than a contiguous gene deletion including WT1, neither he nor his offspring will be at increased risk for the WAGR syndrome.

## Conclusion

In summary, we identified a 7 year old male with aniridia and congenital fibrosis of the extraocular muscles as a result of independent mutations in the PAX6 and KIF21A genes, inherited independently from his father and mother. The c.2860C > T (p.Arg954Trp) from his mother is a recurrent mutation in the KIF21A and the c.745delC (p.Leu249TyrfsX22) mutation in the PAX6 from his father is a novel mutation which expands the mutation spectrum of PAX6.

## Competing interests

The authors declare that they have no competing interests.

## Authors’ contributions

YM and HR performed genomic sequencing and wrote the manuscript; HP and WL performed the clinical assessment of patients; LN supervised the studies and critically revised the manuscript. All authors read and approved the final manuscript.

## Pre-publication history

The pre-publication history for this paper can be accessed here:

http://www.biomedcentral.com/1471-2350/14/63/prepub
